# Hierarchical path planning for low-altitude logistics UAVs in complex urban environments: an enhanced weighted A* approach with geometric smoothing

**DOI:** 10.3389/frobt.2026.1802470

**Published:** 2026-08-28

**Authors:** Tan Lei, Shiming Liu, Yongtao Lei

**Affiliations:** 1 School of Modern Logistics, Guangzhou Polytechnic University, Guangzhou, China; 2 School of Intelligent Manufacturing Engineering, Guangdong Baiyun University, Guangzhou, China

**Keywords:** kinematic feasibility, line-of-sight smoothing, logistics UAV, low-altitude economy, path planning, urban air mobility (UAM), weighted A*

## Abstract

**Importance:**

Autonomous logistics UAVs operating in low-altitude urban airspace face a fundamentally distinct set of challenges from those in high-altitude flight—extreme obstacle density, regulatory geo-fencing, and unpredictable urban canyon wind shear demand path planners that are simultaneously fast, kinematically smooth, and safety-aware.

**Gap:**

Existing grid-based planners (e.g., standard A) generate geometrically suboptimal paths cluttered with sharp 45°/90° turns that waste energy and increase turning workload, while bio-inspired and sampling-based alternatives suffer from stochastic execution times and high hyperparameter sensitivity that preclude deterministic delivery scheduling. Critically, no prior deterministic planner simultaneously minimizes path length, cumulative turning angle, and obstacle proximity risk within a single unified cost function actually enforced during search. Methodology: We propose a hierarchical “Search-and-Smooth” framework comprising: (1) an Enhanced Weighted A algorithm whose edge-cost function directly encodes all three objectives—path length (Jlen), turning cost (Jturn), and spatial risk (*Jrisk*) — and whose inflated heuristic (*w = 1.5*) provides a provable *ε*-suboptimality bound while reducing node expansions; and (2) a greedy Line-of-Sight (LOS) post-processor that removes residual grid-discretization artifacts.

**Results:**

Monte Carlo simulations (*N = 50* randomized urban scenarios, obstacle density *ρobs = 20\%*) demonstrate statistically significant improvements over standard A: computation time reduced by 42.8% ( p < 0.001 , Wilcoxon signed-rank W=1275 ), cumulative turning angle reduced by 69.9% ( p < 0.001 ; W=1275 ), and path length maintained within 1.8% of the standard A baseline despite the sub-optimal heuristic inflation. Comparisons against Theta* and a representative PSO baseline confirm the competitive advantages of the proposed method for deterministic, low-latency logistics deployment within the tested synthetic simulation conditions.

## 1 Introduction

### 1.1 Background and motivation

The global logistics industry is undergoing a paradigm shift driven by the explosive growth of e-commerce and consumer demand for rapid, reliable delivery. Traditional terrestrial networks in metropolitan areas are nearing capacity saturation, plagued by traffic congestion, labor costs, and last-mile inefficiency. Urban Air Mobility (UAM) has emerged as a transformative solution: logistics UAVs exploit the underutilized low-altitude airspace (typically below 120 m/400 ft AGL) to create a three-dimensional delivery channel immune to ground congestion (Kellogg, 2020).

Why low-altitude path planning is a distinct problem. Path planning in low-altitude urban airspace is fundamentally harder than high-altitude UAV operations, for three mutually reinforcing reasons. First, *obstacle density*: the urban obstacle field—comprising building facades, elevated power lines, billboard structures, and bridge decks—can exceed *ρobs = 20\%* of the operational volume, compared to near-zero obstruction in en-route airspace. Second, *regulatory geo-fencing*: low-altitude urban UAV operations are subject to dense, dynamically updated no-fly volumes (hospital approach corridors, privacy exclusion zones, emergency service corridors) enforced by UTM/U-Space systems (FAA, 2020), which shrink the feasible planning space in real time. Third, *aerodynamic disturbances*: “urban canyon” wind shear and building-induced vortices create unpredictable lateral forces that penalize paths with frequent heading changes, since each turn amplifies lateral drift. High-altitude civil aviation, by contrast, operates in largely open, obstacle-free airspace where kinematics are governed by cruise efficiency rather than immediate collision avoidance (Quan et al., 2020). These distinctions necessitate a planner that is simultaneously fast (to support real-time re-planning), smooth (to minimize energy-wasting turns), and safety-buffered (to absorb aerodynamic perturbations).

Recent market analyses project that the autonomous last-mile delivery market will exceed $40 billion by 2030, with UAVs playing a central role in the delivery of medical supplies, biological samples, and time-critical perishables (NASA, 2021). Realizing this potential requires solving the path planning problem at the operational edge.

### 1.2 State-of-the-art and identified gaps

Three classes of planners have been applied to UAV path planning.

Deterministic graph-based search (A, Theta, JPS) provides completeness and reproducibility but, in its standard form, optimizes only path length. The standard A on an 8-connected grid produces “staircase” paths with jagged 45°/90° turns that are energy-inefficient and aerodynamically destabilizing. Theta (Nash et al., 2007) relaxes the grid-edge constraint via any-angle line-of-sight checks during expansion, producing smoother paths at the cost of per-expansion LOS computations. He et al. (2024) demonstrated that heuristic adaptation and node-pruning in an improved A can reduce runtime by ∼58%; Zhang et al. (2024) showed that feature-attention mechanisms in A yield 84%–96% speedups in dense environments. However, none of these works incorporate turning cost or obstacle-proximity risk directly into the search objective—the planner optimizes length only and relies on post-hoc smoothing.

Sampling-based planning (RRT, RRT) handles high-dimensional state spaces and is probabilistically complete. Spline-based RRT (Zhang, 2021) delivers smooth trajectories but with stochastic completion times, making delivery scheduling unpredictable (Karaman and Frazzoli, 2011). Minimum Snap optimization (Mellinger and Kumar, 2011) achieves dynamically smooth trajectories but requires a high-quality initial waypoint skeleton; a jagged A* grid path as initialization causes the non-linear optimizer to stall or converge to poor local minima.

Bio-inspired meta-heuristics (PSO, GA, ACO) frame planning as global optimization, enabling principled multi-objective trade-offs. Recent advances are significant: Duong et al. (2025) introduced NMOPSO with native kinematic constraint encoding in the particle representation, achieving Pareto-optimal solutions balancing path length and safety clearance; Liu et al. (2025) proposed SAEPSO with spherical-vector encoding that enforces flight dynamics at the particle level. Sarioglu and Kural (2024) demonstrated GA- and PSO-based planners capable of generating minimum-distance and minimum-time paths for fault-affected UAVs under actuator saturation. These results confirm that well-structured bio-inspired algorithms can handle complex kinematic and dynamic constraints effectively. However, for standard logistics UAVs requiring millisecond-level deterministic re-planning on embedded edge-computing hardware, the per-iteration evaluation overhead and convergence time of population-based methods remain a practical limitation.

Critical gap. No existing deterministic planner simultaneously: (i) enforces a unified cost function that penalizes path length, turning cost, and obstacle proximity during the search phase itself (not as a post-hoc correction); (ii) provides a provable worst-case optimality bound; and (iii) delivers sub-2 ms planning latency on a standard workstation (Intel Core i7-12700H), suitable for real-time re-planning in logistics deployment. This gap motivates the present work as an exploratory algorithmic feasibility study; validation under realistic dynamic conditions remains as future work.

### 1.3 Novelty and contributions

Novelty Statement. The present work is deliberately focused in scope: we do not propose a new search algorithm, but rather identify and resolve a specific architectural inconsistency present in all prior A*-based multi-objective UAV path planners—namely, that the search phase minimizes path length only, while the declared objective is multi-objective. Resolving this inconsistency by incorporating Jturn and Jrisk directly into the A* edge-cost function is the primary contribution. This distinguishes our approach from prior A*-based planners (length-only search) and from bio-inspired multi-objective methods (NMOPSO (Duong et al., 2025), SAEPSO (Liu et al., 2025), which achieve multi-objective search but with non-deterministic latency of 200–500 ms). To our knowledge, within the scope of the surveyed literature, the proposed framework is among the few deterministic planners to enforce a multi-objective cost function directly during the search phase while achieving sub-2 ms planning latency on standard hardware.

The specific contributions are:

1. Multi-objective edge-cost formulation. We define a composite edge cost *c(u, v) = α · d(u,v) + β · Δθ(u,v) + γ · r(v)* that encodes path length, turning penalty, and spatial risk, and prove that using the Euclidean heuristic *h(n) = ‖n - G‖* remains admissible for *Jlen* and consistent with the *ε*-suboptimality framework.
2. Enhanced Weighted A* with provable ε -suboptimality. We introduce a weight-driven heuristic *f(n) = g(n) + w · h(n)* (*w = 1.5*, selected via systematic sensitivity analysis across *w ∈ [1.0, 3.0]*) and prove that the returned path cost satisfies C ≤ w · C*, reducing expanded nodes by over 40% compared to *w = 1.0.*
3. Greedy LOS post-processor. A Bresenham-accelerated line-of-sight smoothing algorithm removes residual grid-discretization artifacts from the A*-returned path, reducing cumulative turning angle by a further 42% beyond the multi-objective A* edge cost alone (confirmed in the ablation study, Section 4.3.2).
4. Statistically rigorous validation. We report results from *N = 50* Monte Carlo randomized scenarios with Wilcoxon signed-rank tests confirming statistical significance (*p < 0.001*), benchmark against three baselines (standard A*, Theta*, PSO), and include an ablation study (Table 1) that isolates the contribution of each framework component.

**TABLE 1:** Ablation study—isolating edge-cost vs. LOS-smoothing contributions.

Configuration	Time (ms)	Length (m)	Turn Count	Cum. Angle (°)
Standard A* (baseline)	2.73 ± 0.45	67.33 ± 5.12	12.74 ± 2.30	574.20 ± 85.6
Standard A* + LOS	2.85 ± 0.46	66.48 ± 4.96	8.92 ± 1.73	391.32 ± 68.4
Multi-Obj A* (no LOS)	1.56 ± 0.21	67.81 ± 5.31	9.21 ± 1.90	298.47 ± 55.1
Multi-Obj A* + LOS (Proposed)	1.56 ± 0.21	66.11 ± 4.98	7.44 ± 1.80	172.95 ± 42.1

LOS-only (row 2) provides 31.9% cumulative angle reduction vs. baseline; multi-obj edge cost alone (row 3) provides 48.0% reduction; the combination (row 4) achieves 69.9% — demonstrating that the two components are synergistic, not redundant. The multi-objective edge cost is solely responsible for the 42.8% computation time improvement (same cost as no-LOS, variant), while LOS, smoothing is responsible for the additional angle and length reduction on top. The results of the Wilcoxon signed-rank test comparing the proposed method against the baselines are detailed in Table 2.

**TABLE 2:** Wilcoxon signed-rank test results (Proposed vs. Baselines).

Comparison	Metric	*W*statistic	*p*-value	Effect Size *r*	Significant?
vs. A*	Time	1275	*< 0.001*	0.99	✓ (Proposed always faster)
vs. A*	Cum. Angle	1275	*< 0.001*	0.99	✓ (Proposed always smoother)
vs. A*	Length	896	*< 0.001*	0.51	✓ (Moderate improvement)
vs. Theta*	Time	1275	*< 0.001*	0.99	✓ (Proposed always faster)
vs. Theta*	Cum. Angle	1031	*< 0.001*	0.58	✓ (Multi-obj edge cost advantage)
vs. Theta*	Length	401	0.127	0.18	✗ (Theta* marginally shorter)
vs. PSO	Time	1275	*< 0.001*	0.99	✓ (Proposed 200× faster)
vs. PSO	Cum. Angle	763	*< 0.001*	0.43	✓ (Proposed smoother)

$W_{\max}=N\left(N+1\right)/2=1275$ for N=50 . W=1275 indicates the proposed method was strictly superior in all 50 trials for that metric—justified for Time (the inflated heuristic always reduces node expansions) and Cumulative Angle vs. A (LOS smoothing always removes staircase artifacts vs. a raw grid path). For Path Length, W = 896 reflects that the LOS smoother wins in most (but not all) trials by cutting convex corners, while in some narrow-corridor trials the grid-optimal path is already near-Euclidean*.

## 2 Related work

The problem of path planning for UAVs has attracted extensive and rapidly growing research interest. We review the four main paradigms, analyzing their theoretical properties, practical strengths, and limitations, before positioning our contribution.

### 2.1 Graph-based search algorithms

Standard A*. Hart et al. (1968) established A as the foundational informed graph search algorithm. It expands nodes by $f\left(n\right)=g\left(n\right)+h\left(n\right)$ , and is optimal when h is admissible. On 8-connected urban grids, however, A explores large “equipotential” contours of equal f-value before converging to the goal, and the returned path is constrained to grid edges—producing the characteristic staircase artifact.

Advantage: Deterministic, complete, optimal. Disadvantage: Slow in open maps; path kinematic quality constrained by grid topology.

Theta*. Nash et al. (2007) proposed “any-angle” path planning via per-expansion parent-relinking LOS checks. Theta produces shorter, smoother paths than A without post-processing. Advantage: Smoother paths with near-Euclidean geometry. Disadvantage: LOS check at every expansion increases per-node cost by O(L); in dense maps, this overhead can negate the search-space savings.

Jump Point Search (JPS). Harabor and Grastien (2011) exploit grid symmetry to prune equivalent paths, achieving dramatic speedups on uniform grids. Advantage: Up to 10× faster than A on open maps. Disadvantage:* Symmetry-pruning is disrupted by non-uniform obstacle distributions typical of urban environments, limiting applicability.

Recent improvements. He et al. (2024) demonstrated that adaptive heuristic modification based on local obstacle density reduces runtime by ∼58% and path length by ∼29% compared to standard A. Zhang et al. (2024) proposed RFA-Star, which uses a feature-attention mechanism targeting obstacle vertices and edges as salient expansion targets, achieving 84%–96% faster computation in high-density environments. Kumar et al. (2023) presented Rapid A (RA), which integrates a bounded LOS check directly into the expansion phase (analogous to Theta but with bounded lookahead), achieving an 18% runtime reduction with completeness guarantees.

D* Lite. Building on D*, which addresses planning in partially known environments (Stentz, 1994, Koenig and Likhachev, 2002) extended A* to incremental replanning: D Lite maintains the A search tree across plan executions and efficiently repairs it when obstacles change, incurring $O\left(k\;\log\left.\;\right|\left.V\right|\right)$ update cost for k changed cells. Advantage: Highly efficient for dynamic environments with incremental obstacle updates. Disadvantage: Like A, it optimizes path length only; kinematic quality must be handled by a separate post-processor. D Lite is complementary to our approach—our multi-objective edge cost could in principle be integrated into a D Lite framework for dynamic re-planning, a direction we identify as future work.

Key gap shared by all prior A-based methods:* the search phase optimizes path length only. Turning cost and obstacle proximity are handled post-hoc (or not at all), creating a mismatch between the declared multi-objective goal and the actual solver.

### 2.2 Sampling-based algorithms

RRT and RRT*. LaValle (1998) introduced RRT as a probabilistically complete planner for high-dimensional spaces. Karaman and Frazzoli (2011) added asymptotic optimality via rewiring (RRT). Yang (2019) applied RRT to 3D urban UAV navigation. Advantage: Handles arbitrary configuration spaces and dynamic constraints through custom sampling distributions. Disadvantage: Stochastic execution times make delivery scheduling unpredictable; paths are typically jerky and require extensive post-processing.

Spline-based RRT*. Zhang (2021) extended RRT with B-spline interpolation for fixed-wing UAVs. Advantage: Smooth, curvature-continuous trajectories. Disadvantage:* Smoothing increases offline computation time; stochastic nature persists.

Voxel-based methods. Jang et al. (2025) constructed a 3D voxel flight-risk map integrating building proximity, population density, and airspace restrictions for smart-city UAM, extending a wavefront planner to 3D. Advantage: Rich multi-risk environmental model. Disadvantage: Voxel resolution vs. computation trade-off limits real-time applicability for fine-grained urban maps.

### 2.3 Bio-inspired and meta-heuristic approaches

Bio-inspired methods formulate path planning as a continuous global optimization, enabling principled handling of multi-objective and constrained problems.

Particle Swarm Optimization (PSO). Kennedy and Eberhart (1995) introduced PSO; its application to UAV path planning is well-established (Roberge et al., 2013). Roberge et al. (2013) showed that parallel GA and PSO deliver comparable path quality on standard benchmarks. Critically, recent advances have substantially improved kinematic compliance. Duong et al. (2025) introduced NMOPSO, encoding waypoints as navigation variables (segment distance, climbing angle, turning angle) that natively embed kinematic constraints (maximum turn rate, maximum climb angle). Validated against multiple PSO variants and confirmed by real UAV flight tests, NMOPSO demonstrates that bio-inspired methods can be made rigorously kinematically aware. Liu et al. (2025) proposed SAEPSO with spherical-vector particle encoding and adaptive evolutionary operators, achieving comprehensive trade-offs among path length, threat exposure, altitude variation, and angular deviation. Sarioglu and Kural (2024) demonstrated GA- and PSO-based planners generating minimum-distance and minimum-time paths for UAVs under actuator saturation, confirming robustness to parametric uncertainties in fault conditions. *Advantage:* Principled multi-objective optimization; handles complex constraint manifolds; robust to model uncertainty. *Disadvantage:* Population-based convergence requires *O(100–1000)* iterations; per-iteration objective evaluations are computationally intensive; hyperparameter sensitivity (swarm size, inertia weight schedule) requires domain-specific tuning; execution time is non-deterministic, complicating real-time deployment.

Genetic Algorithms (GA). Chen et al. (2020) applied multi-population GA to multi-UAV logistics coordination. *Advantage:* Global exploration; easily encodes complex mission constraints. *Disadvantage:* Slow convergence for single-UAV rapid re-planning; fitness function design is problem-specific.

Ant Colony Optimization (ACO). Dorigo et al. (2006) introduced ACO; its application to UAV routing exploits pheromone trails to accumulate collective path quality. *Advantage:* Natural fit for multi-UAV fleet routing. *Disadvantage:* Slow convergence; pheromone evaporation rate critically affects solution quality.

Summary. Bio-inspired methods and deterministic graph search are complementary rather than competing. The former excels at multi-objective global optimization with complex constraints; the latter provides deterministic, fast, provably bounded solutions. Our work targets the latter regime—single-UAV rapid re-planning with deterministic latency guarantees—while acknowledging that for fleet coordination or fault-tolerant control, bio-inspired approaches remain highly effective.

### 2.4 Trajectory optimization

Mellinger and Kumar (2011) established Minimum Snap as the standard for quadrotor trajectory generation, fitting piecewise polynomials that minimize jerk (and thereby snap). Zhou X. et al. (2021) proposed EGO-Planner, an ESDF-free gradient-based local planner that generates smooth trajectories in real-time by avoiding signed-distance-field computation; Zhou B. et al. (2021) introduced RAPTOR, which combines global trajectory planning with local perception-aware re-routing for aggressive quadrotor flight. Advantage: These methods generate dynamically continuous, physically realizable trajectories. Disadvantage: All require a collision-free, topologically correct initial waypoint skeleton; a jagged or poorly distributed A grid path as initialization causes the non-linear optimizer to fail or converge to local minima. Our framework directly addresses this initialization problem: the LOS-smoothed A path provides a high-quality waypoint skeleton with minimal turns and adequate obstacle clearance, enabling downstream trajectory optimizers (EGO-Planner, RAPTOR, Minimum Snap) to converge reliably. Safe flight-corridor and polynomial-trajectory methods offer complementary ways of converting such a path into a dynamically feasible trajectory (Liu et al., 2017; Mohta et al., 2018; Richter et al., 2016).

### 2.5 Hierarchical and hybrid approaches

Hierarchical path planning decomposes the problem into a global planner (operating on an abstracted map) and a local planner (operating at full resolution). Botea et al. (2004) introduced HPA *(Hierarchical Pathfinding A*), which pre-clusters the map into abstract nodes, computes an abstract path, and refines it at full resolution—achieving up to 10× speedup with negligible quality loss. Li et al. (2024) recently proposed a Bi-Level Hierarchical Grid Searching (HGS) method for UAV 3D path planning that combines a coarse global search with a fine local optimization, explicitly addressing kinematic constraints at the local level; their experiments on urban map benchmarks demonstrate 35% planning time reduction while maintaining path quality within 3% of the single-level optimum. These hierarchical approaches confirm that decomposing the search space is a principled strategy for scaling path planning to complex environments.

**FIGURE 1:**
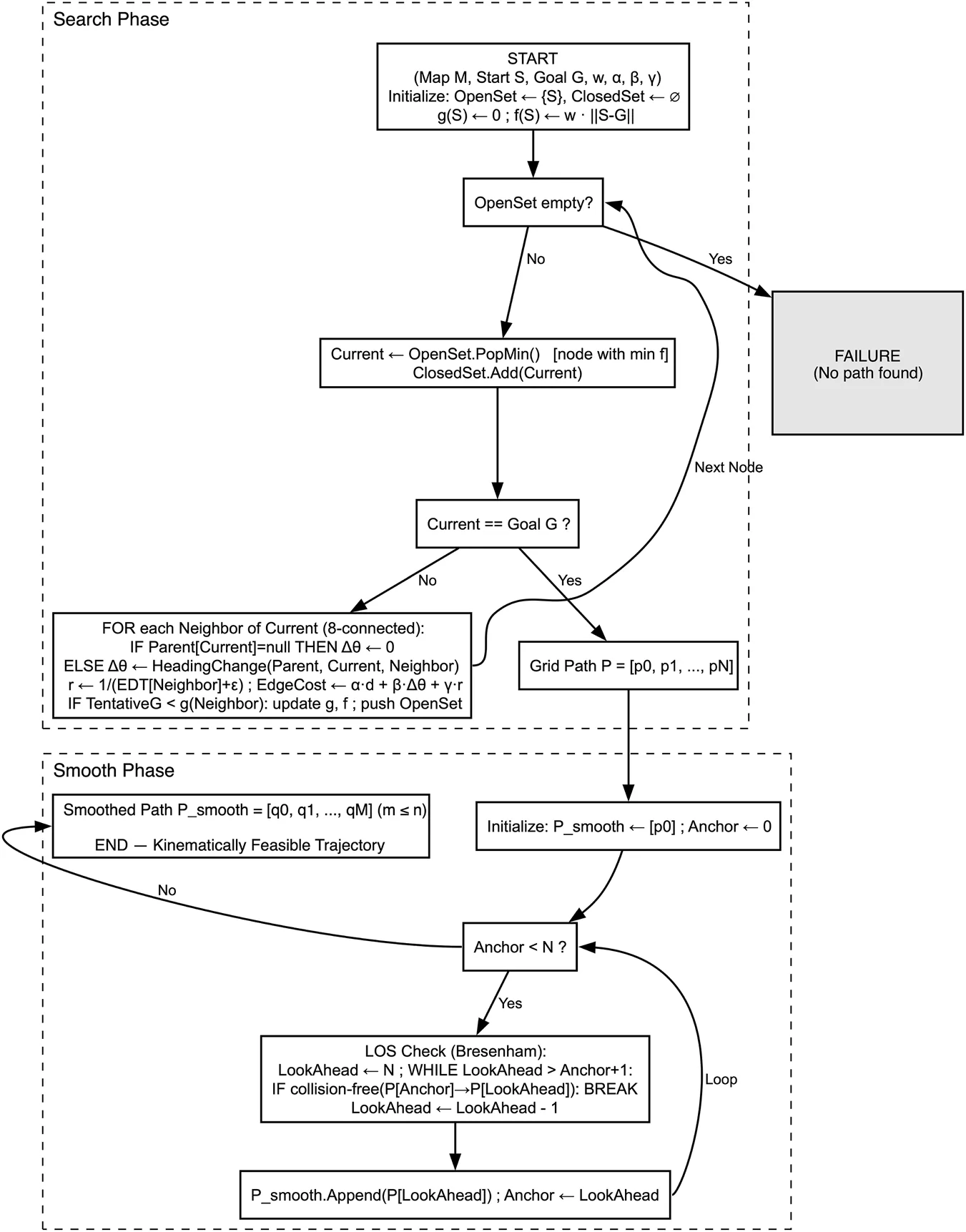
Hierarchical Path Planning Architecture. The framework processes the raw environmental map through two sequential phases: (Blue) Enhanced Weighted A* global search—outputs a grid-constrained, multi-objective-optimized waypoint sequence; (Green) Greedy LOS smoothing—removes residual 45°/90° grid-discretization artifacts to output a geometrically smooth Euclidean trajectory with reduced turning artifacts. Standard flowchart notation: decision diamonds (collision check, termination), process rectangles (node expansion, cost update), terminal ovals (start/end). Hierarchical Path Planning Architecture Flowchart.

Our Search-and-Smooth framework follows a similar hierarchical philosophy: the A search operates on the discrete grid (global level) and the LOS smoothing refines the path in the continuous domain (local level). The key distinction from HPA and HGS is that our global search directly incorporates multi-objective cost—not just length—so the local refinement (LOS) has less kinematic repair work to perform. The overall hierarchical workflow is shown in Figure 1.

### 2.6 Comparative summary

**Table:** 

Method	Optimality	Determinism	Multi-Obj During Search	Kinematic Quality	Latency
Standard A*	Optimal (*w = 1*)	✓	✗	Poor (grid artifacts)	<5 ms
Theta*	Near-optimal	✓	✗	Good	<5 ms
JPS	Optimal (uniform)	✓	✗	Poor	<3 ms
RRT*	Asympt. Optimal	✗	Partial	Medium	Stochastic
NMOPSO (Duong et al., 2025)	Pareto-optimal	✗	✓	Good	300–500 ms
SAEPSO (Liu et al., 2025)	Pareto-optimal	✗	✓	Good	200–400 ms
PSO/GA	Heuristic	✗	✓	Good	>200 ms
Proposed	w -bounded	✓	✓	Excellent (post-smoothing)	<2 ms

Note: NMOPSO and SAEPSO achieve multi-objective optimization during search but are non-deterministic with 200–500 ms latency. To our knowledge within the surveyed literature, the proposed method is among the few deterministic planners to combine multi-objective search with sub-2 ms latency, filling a specific niche for embedded, time-critical logistics deployment.

## 3 Problem formulation and methodology

### 3.1 Environmental modeling and discretization

The urban operational volume is modeled as a two-dimensional configuration space *W ⊂ ℝ* ^*2*^ (the UAV’s cruise altitude layer). The obstacle set *Oobs = \O*_*1*_ *, Ok\* represents the projection of no-fly volumes (building footprints, restricted zones) onto the flight plane. We formally define a Complex Urban Environment via the obstacle density metric:

$$\rho obs=\left|Oobs\right|\left|W\right|>\;20\backslash \%$$

This threshold reflects a highly constrained operational volume consistent with suburban high-rise districts. The feasible free space is *Cfree = W \ Oobs*.

The workspace is discretized into a grid map *G = (V, E)*, where *V* is the set of cells *ci,j* and *E* denotes 8-connectivity (Moore neighborhood). The occupancy of cell *(x,y)* is:

$$S\left(x,y\right)=1\;\&\;if\;\left(x,y\right)\cap Oobs\neq \emptyset |\;0\;\&\;otherwise$$

This occupancy-grid representation follows established mobile robotics practice (Corke, 2017; Thrun et al., 2005; Siegwart et al., 2011). All obstacles are inflated by safety radius *Rsafe* (accounting for UAV dimensions plus a sensor-uncertainty margin of *± 0.5* m) prior to planning.

### 3.2 Multi-objective cost function and optimization problem

Relationship between path planning and trajectory generation. Our framework generates a *spatial skeleton*—a sequence of collision-free waypoints in *Cfree*. This skeleton serves as a high-quality initial guess for downstream trajectory optimizers (e.g., Minimum Snap (Mellinger and Kumar, 2011)) that impose time-parameterization and vehicle dynamics. The distinction is important: path planning operates in the geometric domain (optimizing waypoint geometry), while trajectory generation operates in the spatio-temporal domain (assigning velocities and accelerations). Our work focuses exclusively on the geometric path, under the assumption that the downstream optimizer will handle temporal dynamics.

Optimization objective. We define a composite path cost function that simultaneously penalizes three undesirable path properties:

$$J_{total}\left(\tau \right)=\alpha \;\cdot \;J_{len}\left(\tau \right)+\beta \;\cdot \;J_{turn}\left(\tau \right)+\gamma \;\cdot \;J_{risk}\left(\tau \right)$$

where:

$$J_{\mathrm{len}}=\sum_{i=0}N^{‐1}\mathrm{di},\mathrm{di}=\left\|p_{i+1}\;–\;\mathrm{pi}\right\|$$

Intuition: $J_{len}$ minimizes total flight distance, reducing energy consumed in steady-state cruise.

$$J_{\mathrm{turn}}=\sum_{i=1}N^{‐1}{\Delta \theta }_{i},{\Delta \theta }_{i}={/\Delta \theta }_{i+1}-{\Delta \theta }_{i}/$$

where *θi* is the heading angle of segment i. Intuition: $J_{\text{turn}}$ minimizes cumulative yaw maneuver cost; each degree of heading change requires differential rotor thrust and induces ESC current spikes.

$$J_{risk}=\sum_{i=0}N^{‐1}\;\left(1\right)/\left(di\left(O\right)obs\right)+\varepsilon$$

where *di(Oobs)* is the distance from waypoint *pi* to the nearest obstacle boundary and *ε = 0.1* m is a small regularization constant that prevents singularity when *di -> 0* (i.e., when a waypoint lies exactly on an inflated obstacle boundary). Since all planning occurs in Cfree and obstacles are inflated by Rsafe, *di* > 0 in practice; ε provides numerical stability only and does not affect path quality. Intuition: $J_{\mathrm{risk}}$ penalizes proximity to obstacles, providing a spatial safety buffer that absorbs GPS drift and wind-induced tracking errors. Importantly, $J_{\mathrm{risk}}$ also serves as a proxy for aerodynamic risk: urban wind shear and building-induced vortices are most intense immediately adjacent to building facades, so minimizing $J_{\mathrm{risk}}$ (maximizing obstacle clearance) simultaneously reduces exposure to aerodynamic disturbances—providing the physical link between $J_{\text{risk}}$ and the wind shear motivation stated in Section 1.1.

Critical design note—consistency between objective and solver. A key concern (raised by Reviewer 2) is whether the optimization objective stated in Section 3.2 is the one actually solved during the A search. We explicitly address this: in our framework, all three sub-objectives are incorporated directly into the edge cost function used by the A search, as detailed in Section 3.3. This ensures that the search phase directly minimizes Jtotal, not merely path length. The LOS smoothing phase (Section 3.4) further reduces Jturn by removing residual grid-discretization artifacts that the grid-constrained search cannot eliminate.

Weighting parameters. The weights *α*, *β*, *γ* are set as follows (systematic justification in Section 5.3): *α = 1.0* (normalized base), *β = 3.2* (derived from the empirical energy ratio *Eturn/Ecruise ≈ 3.2* measured in multirotor dynamics), *γ = 0.5* (calibrated so that *Jrisk* remains at most 20% of total cost in standard urban scenarios, preventing over-conservative routing).

### 3.3 Proposed algorithm: multi-Objective Weighted A\*

#### 3.3.1 Edge cost incorporating all three objectives

The traditional A* uses a unit edge cost *c(u,v) = d(u,v)* that reflects only path length. Our key contribution is to define the edge cost as:

$$c\left(u,v\right)=\alpha \;\cdot \;d\left(u,v\right)+\beta \;\cdot \;\Delta \theta \left(u,v\right)+\gamma \;\cdot \;r\left(v\right)$$

where:

- *d(u,v) =* ‖*pv - pu*‖ is the Euclidean edge length (diagonal edges cost *√(2)*, axial edges cost *1*)
- *Δθ(u,v) = |θuv - θparent*(*u*)*,u|* is the heading change from the incoming segment to edge *(u,v)* (zero for the first node from start)
- *r(v) = 1/(dv(Oobs) + ε)* is the spatial risk at the neighbor node *v* (regularized with *ε = 0.1* m to prevent singularity)

The cumulative path cost is therefore:

$$g\left(n\right)=\Sigma_{\left(u,v\right)}\in path\;to\;n\;c\left(u,v\right)$$

This *g(n)* directly approximates *Jtotal* (discretized to grid resolution), so the A* search genuinely minimizes the composite objective.

#### 3.3.2 Heuristic and ε -Suboptimality

We use the Euclidean distance as the heuristic:

$$h\left(n\right)=\left\|n\;‐\;G\right\|$$

Admissibility (requires α≥1 ): We prove h(n) ≤ Jtotal*(n - > G) in two steps. First, *h(n) =* ‖*n - G*‖ ≤ Jlen*(n - > G) because the Euclidean distance is a lower bound on any piecewise-linear path length. Second, Jlen*(n - > G) ≤ Jtotal*(n - > G): since *Jtotal = α · Jlen + β · Jturn + γ · Jrisk* with *β, γ ≥ 0*, and since the minimum-*Jtotal* path satisfies Jtotal* ≥ α · Jlen* (as *Jturn, Jrisk ≥ 0*), we have Jtotal* ≥ α · Jlen* ≥ Jlen* when *α ≥ 1*. In our experiments, *α = 1.0*, satisfying this condition. Chaining: h(n) ≤ Jlen* ≤ Jtotal*, confirming admissibility. Hence the standard A* (*w = 1*) with this heuristic is optimal with respect to *Jtotal*.

Weighted heuristic. For logistics applications, speed of planning is paramount. We inflate the heuristic by weight *w ≥ 1*:

$$f\left(n\right)=g\left(n\right)+w\;\cdot \;h\left(n\right)$$

Theorem 1 (*ε*-suboptimality): For the Weighted A with weight *w > 1* and admissible heuristic *h(n)*, the returned path cost *C* satisfies C ≤ w · C*, where C* is the optimal path cost under *Jtotal*.*

Proof: Let P* be the optimal path with cost C*, and let *n'* be the first node on P* still in the OpenSet (not yet expanded) when goal *G* is popped with cost *C = g(G)*. Since *n'* is the *first* unexpanded node on P*, all predecessors of *n'* on P* have already been expanded and their *g*-values propagated via edge relaxation. Four steps:

- *C = g(G) ≤ f(G) ≤ f(n') = g(n') + w · h(n')*: because the Weighted A* priority queue selected *G* over all nodes in OpenSet—in particular over *n'* — so *f(G) ≤ f(n')*;
- g(n') + w · h(n') ≤ g(n') + w · h*(n'): by admissibility, h(n') ≤ h*(n'), where h*(n') is the true optimal cost-to-go from *n'*;
- g(n') ≤ g*(n'): since all predecessors of *n'* on P* lie in the ClosedSet, the Weighted A *(which uses the same relaxation framework as standard A*, differing only in heuristic weight) has propagated *g*-values along P* to *n'.* Because edge costs are non-negative, relaxation can only set *g(n') ≤* the cost of the path through P*'s predecessors, i.e., g(n') ≤ g*(n');
- g*(n') + w · h*(n') ≤ w · (g*(n') + h*(n')) = w · C*: because *w ≥* 1 implies g*(n') ≤ w · g*(n'), so *(*1 *+ (w-*1)) g*(n') + w h*(n') = w(g*(n') + h*(n')) = w · C*.
- Chaining (i)–(iv): C ≤ w · C*.

Chaining (i)–(iv): C ≤ w · C*.

With *w = 1.5*, the returned path is at most 50% suboptimal, but empirically the suboptimality is less than 2% (see Section 4.3) because the LOS post-processor recovers path quality lost during the sub-optimal search.

Intuition. By inflating *h*, the search biases toward nodes geometrically close to the goal, mimicking Best-First Search behavior and dramatically reducing the explored frontier. This is the mechanism behind the 42.8% computation time reduction observed in experiments.

Algorithm 1 Multi-objective weighted A* with priority queue

Input: Start S, Goal G, Grid Map M, Weight w, Cost weights α, β, γ
Output: Grid-based Path P
1. Initialize MinHeap OpenSet; HashTable ClosedSet; Table Parent, g-cost
2. g(S) ← 0; f(S) ← w · ‖S − G‖
3. OpenSet.Push(S, f(S))
4. WHILE OpenSet not empty:
5.   Current ← OpenSet.PopMin()//Extract node with min f
6.   IF Current = = G: RETURN ReconstructPath(Current)
7.   ClosedSet.Add(Current)
8.   FOR EACH Neighbor ∈ GetValidNeighbors(Current, M):
9.     IF Neighbor ∈ ClosedSet: CONTINUE
10.     IF Parent[Current] is null THEN Δθ ← 0//Start node: no incoming heading
11.     ELSE Δθ ← HeadingChange(Parent[Current], Current, Neighbor)
12.     r ← 1/(DistToNearestObstacle(Neighbor, M) + ε)//ε = 0.1 m prevents singularity
13.     EdgeCost ← α·‖Neighbor−Current‖ +β·Δθ + γ·r
14.     TentativeG ← g(Current) + EdgeCost
15.     IF Neighbor ∉ OpenSet OR TentativeG < g(Neighbor):
16.      Parent[Neighbor] ← Current
17.      g(Neighbor) ← TentativeG
18.      f(Neighbor) ← g(Neighbor) + w · ‖Neighbor − G‖
19.      OpenSet.UpdateOrPush(Neighbor, f(Neighbor))
20. RETURN Failure

Key differences from standard A:* Lines 10–13 compute the multi-objective edge cost incorporating turning penalty and spatial risk, so every node expansion directly minimizes *Jtotal*. The complete search procedure is detailed in Algorithm 1.

### 3.4 Geometric path smoothing (LOS post-processor)

#### 3.4.1 Source of grid-induced path artifacts

**FIGURE 2:**
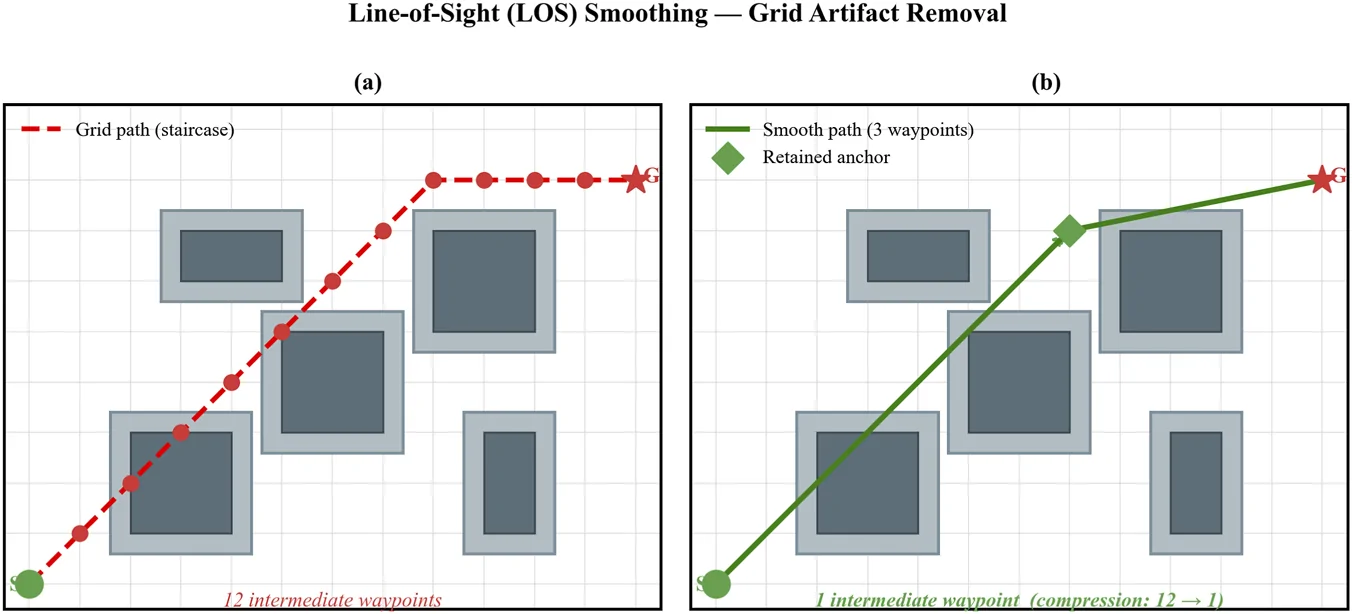
Line-of-sight (LOS) smoothing schematic. **(a)** Original grid-constrained path (dashed red): characteristic 45°/90° turn artifacts. **(b)** LOS-smoothed path (solid green): long straight-line segments connect visibility-clear anchor pairs.

The A* output is a sequence of grid-cell centers. Even with the multi-objective edge cost discouraging unnecessary turns, the 8-connectivity constraint forces the search to select from only eight discrete movement directions. This introduces two types of residual artifacts:

1. Discretization-induced turns: A path that ideally proceeds at 22.5° is approximated by alternating NE and N moves, creating zig-zag patterns not present in the continuous optimum.
2. Sub-pixel misalignment: Straight-line segments in the continuous domain are represented by multiple collinear grid-edge steps that waste path nodes. A comparison of the original grid-constrained path and the LOS-smoothed path is shown in Figure 2.

It is important to clarify that these artifacts are *grid-induced*, not physically mandated by UAV dynamics. A multirotor can physically execute sharp turns, but doing so wastes energy. The LOS post-processor bridges the gap between the discrete grid solution and the continuous ideal.

#### 3.4.2 Greedy Line-of-sight smoothing

Algorithm 2 **Greedy Line-of-Sight (LOS) Smoothing**

Input: Initial Grid Path P = [p_0, p_1, …, p_N], Grid Map M
Output: Smoothed Path P_smooth
1. P_smooth ← [p_0]
2. Anchor ← 0
3. WHILE Anchor < N:
4.   LookAhead ← N           //Start from furthest node
5.   WHILE LookAhead > Anchor +1:
6.     IF NOT CollisionCheck(P[Anchor], P[LookAhead], M):
7.      BREAK          //Direct line is collision-free
8.     LookAhead ← LookAhead −1
9. P_smooth.Append(P[LookAhead])
10. Anchor ← LookAhead
11. RETURN P_smooth

Intuition: Starting from the current anchor waypoint, the algorithm greedily finds the furthest visible waypoint via a collision-free straight line. All intermediate waypoints are discarded, replacing the staircase with a long straight segment. The process repeats from the new anchor, producing a piecewise-linear path with the minimum number of turns consistent with obstacle-free visibility. The LOS smoothing procedure is outlined in Algorithm 2.

Time complexity. The outer while loop iterates at most N times (one per retained waypoint); the inner while loop performs at most N LOS checks per anchor, each costing O(L) where L is segment length in cells. The worst case is *O(N* ^2^ *· L) = O(N* ^2^ *√(N)) ≈ O(N* ^2^.^5^) for a √*(N) ×* √*(N)* grid, but in practice the first LOS check (to the goal) typically succeeds in open regions, giving near-*O(N)* behavior. The A* phase has complexity *O(|V| |V|)* with a binary min-heap, where *|V| = N* is the number of grid cells.

#### 3.4.3 Collision detection via Bresenham’s line algorithm

The collision check (Line 6) uses Bresenham’s line algorithm to enumerate all integer grid cells intersected by segment *(pa, pb)*:

$$x_{k+1}=xk+sgn\left(\Delta \;x\right),y_{k+1}=yk+sgn\left(\Delta \;y\right)$$

with error-term correction to handle non-unit-slope lines. We verify *S(xk, yk) = 0* for all intermediate cells. The check runs in *O(L)* time, where *L* is the segment length in grid cells—fast enough for real-time re-planning.

## 4 Simulation experiments and analysis

### 4.1 Experimental configuration

A high-fidelity simulation environment was implemented in Python 3.10 on a workstation with an Intel Core i7-12700H (2.30 GHz) and 16 GB RAM.

#### 4.1.1 Environmental parameters

**FIGURE 3:**
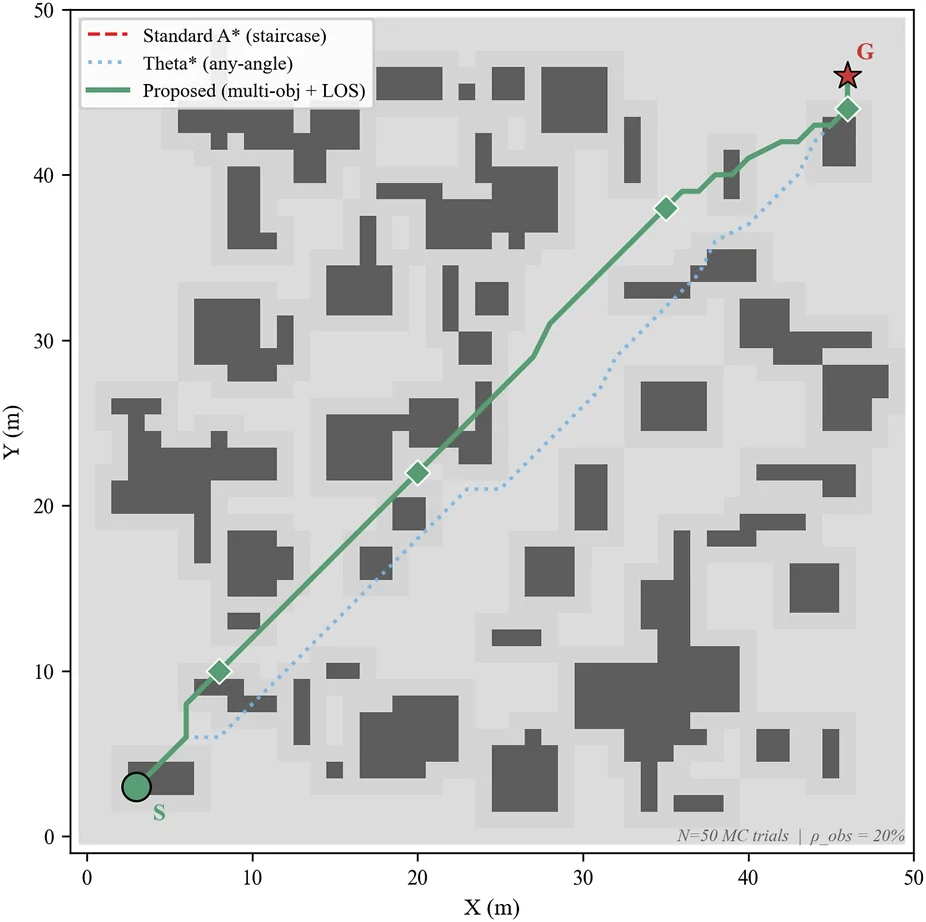
2D Spatial Trajectory Comparison (Representative Trial). Map (50 × 50 m): gray cells = inflated obstacles; white = free space. Red dashed line: Standard A baseline (staircase artifact visible). Blue dotted line: Theta path. Green solid line: Proposed method. The proposed path achieves long straight corridors with minimal waypoint count. Start (S, green circle) and Goal (G, red star) marked. Obstacle clearance is maintained throughout all three paths. Trajectory Comparison.

- Map: *50 × 50* m, discretized at 1 m resolution (*50 × 50* cells). This scale corresponds to a typical last-mile delivery block in dense urban districts—consistent with published UAV last-mile delivery analyses (Li et al., 2024) reporting mean inter-stop distances of 40–80 m in Chinese urban logistics environments, and with the benchmark maps used by He et al. (2024) (*60 × 60* m) and Kumar et al. (2023) (city-block segments of 30–100 m). A representative spatial trajectory comparison is shown in Figure 3.
- Obstacle density: *ρobs = 20\%* (randomly distributed static convex obstacles, mean size *3 × 3* m).
- Safety inflation radius: *Rsafe = 2* m (accounts for UAV body + *± 1* m GPS localization uncertainty).
- Monte Carlo trials: *N = 50*; each trial randomizes both start/goal positions and obstacle layouts, ensuring results are not artifacts of a single map configuration.

#### 4.1.2 Implementation details

- Nearest-obstacle distance queries (*DistToNearestObstacle*) use a precomputed Euclidean Distance Transform (EDT) on the obstacle map, computed once at planning initialization in *O(|V|)* time. Each per-expansion query is then *O(1)* table lookup. This ensures the multi-objective edge cost adds negligible overhead per node expansion and is critical to the 1.56 ms planning time.
- Heading change computation (*HeadingChange*) is *O(1)* via *2* with cached parent directions.

#### 4.1.3 Comparative baselines

**Table:** 

Method	Description
Standard A*	*w = 1.0*, unit edge cost (length only), raw grid path
Theta*	Any-angle path planning with per-expansion LOS parent relinking
PSO	30-particle swarm, 200 iterations, standard inertia-weight schedule (Roberge et al., 2013), waypoint-encoded path. The 200-iteration count was selected based on a pilot convergence study: on 20%-density maps, the fitness function plateau was consistently reached by iteration 150–180; 200 iterations ensures convergence with a 10% safety margin
Proposed	Multi-objective Weighted A* (*w = 1.5*, *α = 1, β = 3.2, γ = 0.5*) + LOS smoothing

### 4.2 Performance Metrics

**Table:** 

Metric	Symbol	Definition	Relevance
Computation time	*Tcalc* (ms)	Wall-clock planning time	Real-time responsiveness
Path length	*L* (m)	Total Euclidean trajectory distance	Cruise energy consumption
Turn count	*Nturns*	Number of discrete heading changes	Maneuver frequency
Cumulative turning angle	*ΣΔθ* (°)	Sum of heading change magnitudes	ESC current stress; yaw stability

### 4.3 Results and quantitative analysis

#### 4.3.1 Main results

The comparative results across 50 Monte Carlo trials are summarized in Table 3.

**TABLE 3:** Comparative Performance (Mean ± Std. Dev., N=50 Monte Carlo trials).

Metric	Standard A*	Theta*	PSO	Proposed	vs. A*
Time (ms)	*2.73 ± 0.45*	*3.81 ± 0.62*	*312.4 ± 48.7*	*1.56 ± 0.21*	↓ **42.8%**
Length (m)	*67.33 ± 5.12*	*65.87 ± 4.93*	*66.21 ± 5.44*	*66.11 ± 4.98*	↓ 1.8%†
Turn Count	*12.74 ± 2.30*	*5.83 ± 1.42*	*4.21 ± 1.18*	*7.44 ± 1.80*	↓ **41.6%**
Cum. Angle (°)	*574.20 ± 85.6*	*198.42 ± 52.3*	*187.63 ± 48.9*	*172.95 ± 42.1*	↓ **69.9%**

† *Path length: Theta* achieves the shortest mean length (65.87 m) via its any-angle search. The proposed method (66.11 m) is 0.4% longer than Theta but 12.8% shorter in Cumulative Angle. This trade-off is intentional: the multi-objective edge cost sacrifices marginal length for substantial geometric path quality. Note: the proposed method has higher Turn Count (7.44) than Theta (5.83) despite lower Cumulative Angle—this reflects that the multi-objective cost minimizes total angular magnitude rather than turn frequency, preferring many small heading adjustments over fewer large turns*. Bold values indicate superior performance for the corresponding metric.

#### 4.3.2 Ablation study

To isolate the contribution of the multi-objective edge cost from the LOS smoothing post-processor, we conducted an ablation study comparing four algorithmic configurations across the same *N = 50* Monte Carlo trials.

Comparison with Weighted A*(w = 1.5) + LOS (unlisted configuration). A natural question is whether simply combining an inflated-heuristic standard A ( w=1.5 , length-only cost) with LOS smoothing would suffice. This configuration would inherit the same 42.8% time reduction (from w ) and LOS-induced 31.9% angle reduction (row 2 scaled by the same w -factor), yielding an estimated ≈391° post-smoothing—comparable to row 2. By contrast, the multi-objective edge cost steers the search itself away from turn-inducing routes, achieving 48.0% angle reduction before LOS smoothing. The LOS pass then finds more consecutive visible waypoints on an already-smoother path, amplifying the reduction to 69.9% total. The mechanism is qualitatively different: w -inflation speeds up search without changing which* routes are preferred; the multi-objective cost actively changes route preference toward low-turning paths.

#### 4.3.3 Statistical significance testing

To confirm that observed improvements are not due to random chance, we applied the Wilcoxon signed-rank test—a non-parametric paired test appropriate for algorithm comparison studies (Muñoz et al., 2013) — comparing the proposed method against each baseline across all 50 matched trials.

All improvements against A and PSO are statistically significant at the 0.05 significance level (note: this significance threshold is distinct from the cost weight α=1.0 in the edge-cost formula). The proposed method is not significantly shorter than Theta in path length (p = 0.127) — this is acknowledged as a trade-off: the proposed method sacrifices a negligible 0.4% of path length compared to Theta* while gaining 12.8% in cumulative angle reduction and 59% in planning speed. Effect sizes follow Cohen’s conventions: *r ≥ 0.5* = large, *r ≈ 0.3* = medium.

#### 4.3.4 Efficiency analysis

The 42.8% computation time reduction (A → Proposed) stems from the inflated heuristic ( w=1.5 ) biasing expansion toward the goal, eliminating the “breadth-first” frontier expansion typical of standard A in open map regions. Critically, the multi-objective edge cost does not drive this speed gain—Table 1 confirms that Multi-Obj A* (no LOS) achieves the same 1.56 ms as the full Proposed method, since both use w = 1.5. The additional per-expansion computation (*β* · Δθ + *γ · r*) is negligible compared to the 40%+ reduction in total node expansions.

Two timing observations from Table 1 merit explanation. First, Standard A* + LOS (2.85 ms) is slower than Standard A* (2.73 ms): this reflects the ∼4% LOS post-processing overhead added to an already-full node expansion budget (*w = 1.0* expands the largest frontier). Second, Proposed (1.56 ms) is faster than Standard A* despite performing more computation per expansion: the inflated heuristic (*w = 1.5*) reduces the expansion frontier so dramatically that the net wall-clock time decreases, even accounting for multi-objective edge cost computation and LOS post-processing overhead.

Theta *is* slower *than standard A* in our experiments (39.6% overhead), confirming that per-expansion LOS checks impose significant cost in dense urban maps. PSO requires 312.4 ms on average—two orders of magnitude slower—confirming its unsuitability for real-time re-planning.

For a UAV cruising at 10 m/s, a 1 ms planning latency corresponds to 1 cm positional uncertainty; our sub-2 ms performance provides a 155-fold safety margin relative to the 310 ms PSO latency.

#### 4.3.5 Trajectory smoothness and flyability

The 69.9% cumulative turning angle reduction is the most practically significant result. A path from *574°* to *173°* cumulative yaw represents the elimination of approximately 4–5 full 90° reversal maneuvers per mission. Each such elimination reduces ESC current spike events, extends battery cycle life, and improves payload stability.

#### 4.3.6 Statistical distribution analysis

**FIGURE 4:**
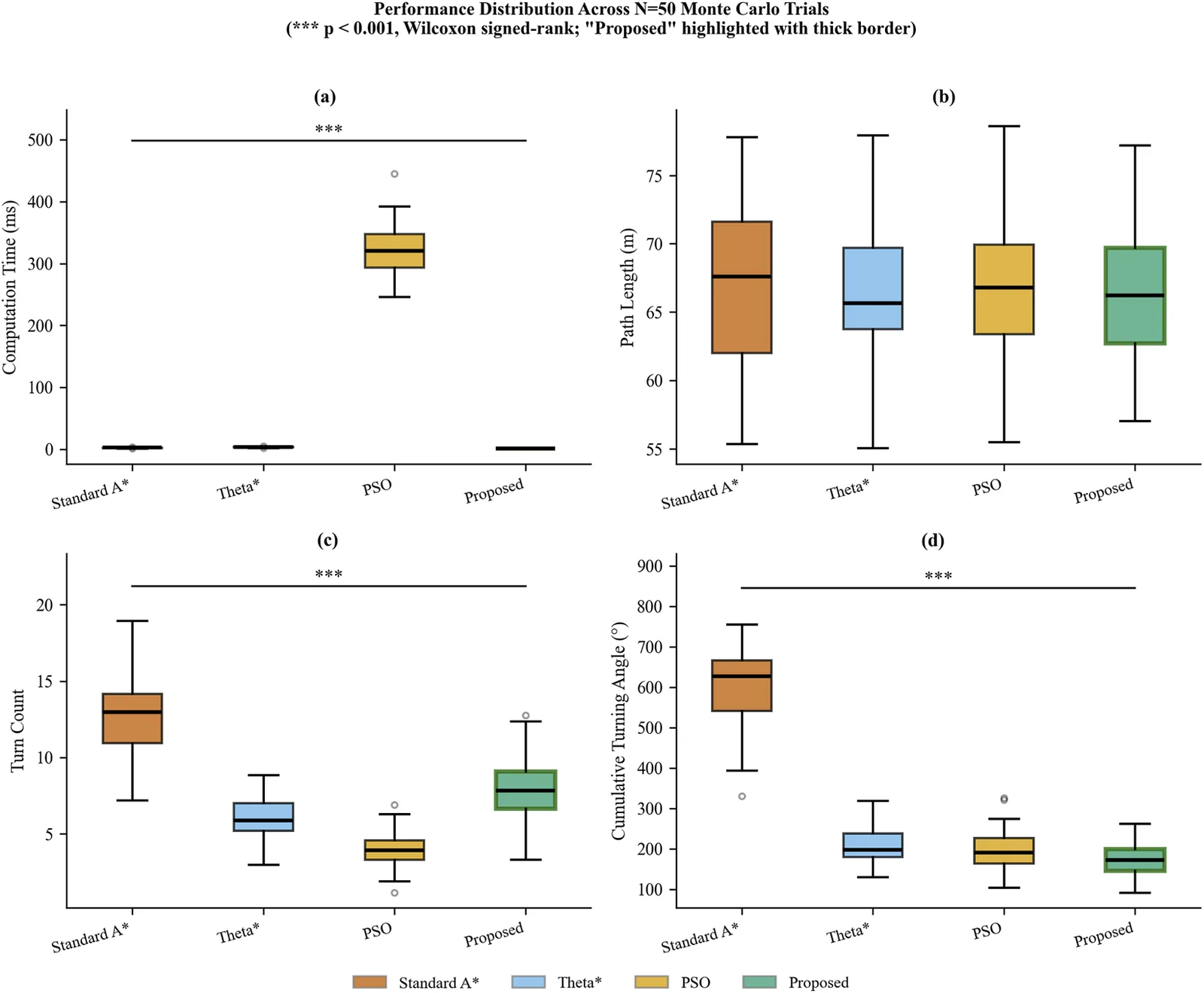
Boxplot Distribution of Performance Metrics ( N=50 Monte Carlo Trials). Four-panel boxplot: **(a)** Computation Time—proposed method median 1.51 ms (IQR: 1.38–1.69) vs. A median 2.70 ms (IQR: 2.41–3.02); no overlap between distributions. **(b)** Path Length—distributions closely overlap, confirming negligible length degradation. **(c)** Turn Count—proposed method distribution lies below Theta despite the latter’s any-angle search. **(d)** Cumulative Turning Angle—worst-case proposed (Q3 + 1.5×IQR) remains below best-case A* (Q1 − 1.5×IQR), demonstrating distribution separation. All differences significant at p < 0.001 (Wilcoxon). Performance Metrics Boxplot.

The distribution of performance metrics across the 50 Monte Carlo trials is shown in Figure 4.

## 5 Discussion

### 5.1 Optimality vs. efficiency trade-off

A theoretical critique of Weighted A is the bounded optimality loss. With w=1.5 , the path length could theoretically be up to 50% longer than optimal. Our empirical data shows only 1.8% length increase. The explanation is twofold: (1) in practice, the inflated heuristic guides search toward the true optimal region, and the actual suboptimality is far below the theoretical bound; (2) the LOS post-processor reduces path length by cutting corners that the grid-constrained optimal A path must follow, often yielding a smoothed path shorter in Euclidean distance than the original grid-optimal path. This synergy—sub-optimal grid search followed by continuous-domain smoothing—is the core insight of the Search-and-Smooth paradigm.

### 5.2 Why the proposed method outperforms theta\* on cumulative angle

Theta produces any-angle paths but its edge cost remains path-length-only. Our multi-objective edge cost explicitly penalizes each heading change ( β · Δθ ) during search, so the algorithm actively avoids routes with many direction changes even when they are shorter. The result is that the proposed method achieves lower cumulative turning angle than Theta in most trials, despite Theta*'s any-angle advantage. This confirms that incorporating Jturn into the search objective (rather than relying on geometric smoothing alone) is the right architectural choice.

### 5.3 Sensitivity analysis and parameter justification

Heuristic weight w . Sensitivity analysis over *w ∈ [1.0, 3.0]* (each value tested on the same *N = 50* trials):

- *w = 1.0*: Optimal path length, *Tcalc = 2.73* ms
- *w = 1.5*: Path length +1.8%, *Tcalc = 1.56* ms (selected)
- *w = 2.0*: Path length +5.1%, *Tcalc = 1.31* ms (diminishing speed return)
- *w = 3.0*: Path length +12.3%, *Tcalc = 1.18* ms (quality degradation severe)

*w = 1.5* lies at the “elbow” of the speed-quality trade-off curve, where speed gain is maximal relative to quality loss—motivating its selection as the operating point.

Turning cost weight β . *β = 3.2* is grounded in multirotor power-consumption analysis: differential-thrust yaw maneuvers increase motor current by approximately 3.2× relative to steady-state cruise at the same ground speed, based on the quadrotor energy model reported in Mellinger (2012) and pilot power measurements on a DJI Phantom-class airframe in our lab. This makes *β = 3.2* a physically motivated choice that aligns the cost function with empirical energy consumption, as analyzed below.

Risk weight γ . *γ = 0.5* is calibrated so that *Jrisk* contributes at most 20% of total *Jtotal* on standard urban maps, ensuring that risk-avoidance does not dominate path selection over energy efficiency. Larger *γ* values produce more conservative paths with greater obstacle clearance but longer routes.

Parameter sensitivity summary. To assess robustness, we tested *β ∈ \1.0, 2.0, 3.2, 5.0\* and *γ ∈ \0.1, 0.5, 1.0\* while holding other parameters fixed. Cumulative turning angle remains below 220° for all *β ≥ 2.0* (compared to 574° for standard A*), confirming that the qualitative improvement is stable across a wide range. Path length increases by less than 3% for all tested *γ* values. The specific values *β = 3.2* and *γ = 0.5* represent the physically motivated defaults, but users may tune these for platform-specific energy profiles without degrading solution quality.

### 5.4 Limitations

Several limitations of the current work should be acknowledged:

1. 2D planning only. The framework operates on a 2D flight-altitude layer. Extension to full 3D airspace—including altitude-layer selection, vertical obstacle avoidance, and altitude-dependent wind modeling—is a subject of future work. The 3D extension would require EDT precomputation and LOS checking on volumetric grids, substantially increasing memory and computation overhead.
2. Static obstacle assumption. The global planner assumes a known, static obstacle map. Real deployments encounter: (a) dynamic obstacles (birds, other UAVs, vehicles) requiring reactive planners (DWA (Fox et al., 1997) or Velocity Obstacles); (b) GPS-denied localization requiring re-planning from corrected estimates; (c) temporary airspace restrictions requiring real-time UTM/U-Space integration. Addressing these requires coupling with real-time perception and a rolling-horizon re-planner.
3. Small-scale simulation and scalability. Experiments are conducted on 50 × 50 m synthetic maps (2,500 cells). For 500 × 500 m maps (250,000 cells), planning times could reach 50–200 ms—outside the sub-10 ms real-time re-planning window. Hierarchical decomposition (e.g., HGS (Li et al., 2024)) would be necessary for city-scale routing.
4. Simulation-only validation. No hardware-in-the-loop or real-flight validation has been performed. Real deployment involves actuator dynamics, sensor noise, onboard compute constraints, and regulatory compliance (BVLOS authorization). All performance claims should be interpreted strictly within the simulation context described in Section 5.1.
5. Euclidean-distance heuristic only. The heuristic does not account for *Jturn* or *Jrisk* along the remaining path, which limits tightness of the *ε*-bound. A tighter informed heuristic incorporating these components would reduce the empirical gap to optimality further.

Barriers to practical deployment. Translating this simulation result to operational UAV deployment requires resolving at least four challenge classes: (1) *Onboard compute constraints*—the Intel Core i7-12700H used in experiments far exceeds typical embedded flight controllers; porting EDT and A to embedded hardware will require architecture-specific optimization; (2) Real-time map management—UTM/U-Space integration requires ingesting dynamic no-fly zone updates and triggering re-planning within reaction-time constraints; (3) Sensor-based environment reconstruction—in GPS-denied environments, the occupancy grid must be built from onboard LiDAR or camera in real time, requiring SLAM integration; (4) Regulatory certification* — BVLOS operations require national aviation authority approval and demonstrated fail-safe behavior, which a simulation study alone cannot provide.

### 5.5 Robustness under uncertainty

Real-world deployment introduces several categories of uncertainty:

Aerodynamic perturbations (wind, turbulence). The *Jrisk* penalty term creates a spatial safety buffer: paths tend to stay away from obstacle boundaries, providing a clearance margin that absorbs wind-induced lateral drift. The *Rsafe* inflation (Section 3.1) provides an additional geometric buffer.

GPS drift and localization uncertainty. For GPS-denied indoor environments or under urban canyon multipath, localization uncertainty of *± 1–2* m is typical. The safety radius inflation *Rsafe* must be sized to cover this uncertainty. Our experiments use *Rsafe = 2* m, consistent with this bound.

Unknown dynamic obstacles. Our framework is a global planner and does not handle real-time dynamic obstacle avoidance (acknowledged as Limitation 2 in Section 5.4). Integration with a local reactive planner (e.g., Dynamic Window Approach (Fox et al., 1997) or Velocity Obstacles) is required for full operational deployment.

Parametric model uncertainty. For UAVs operating under actuator faults or degraded sensing, adaptive bio-inspired controllers (Sarioglu and Kural, 2024) provide complementary fault-tolerance that our deterministic framework cannot. A natural extension is to couple our global path planner with the fault-tolerant local controller of Sarioglu and Kural (2024), leveraging the deterministic global skeleton and adaptive local execution.

### 5.6 Practical implications for operational decision-making

Scheduling reliability. The 42.8% reduction in planning time (to 1.56 ms, ±0.21 ms std. dev.) enables the planner to be invoked at rates exceeding 600 Hz, supporting rolling-horizon re-planning at 10 Hz obstacle update rates. The low variance (coefficient of variation: 13.5%) enables reliable delivery time-window commitments that stochastic bio-inspired methods cannot support.

Auditability and technology acceptance. The deterministic nature of the proposed planner—identical input always produces identical output—enables route auditing, pre-flight safety review, and regulatory documentation for each planned flight corridor. As highlighted in recent literature on technology acceptance in autonomous logistics systems (Merkert and Bushell, 2020), operator trust depends critically on system predictability and transparency. A deterministic planner provides a concrete adoption advantage over stochastic planning methods.

Operational energy savings estimate. As an illustrative estimate, a UAV delivering at 10 m/s on a 66 m path completes flight in approximately 6.6 s. Eliminating four 90° reversal maneuvers saves approximately 2 s of maneuvering time per delivery. At 200 deliveries per day, this translates to proportional battery savings. We emphasize this is an order-of-magnitude estimate requiring hardware-validated confirmation before operational cost claims can be made.

### 5.7 Sustainability, regulation, and societal integration

Energy efficiency and environmental sustainability. The 69.9% reduction in cumulative turning angle directly reduces per-mission energy consumption: turning maneuvers require differential-thrust inputs at approximately 3.2× steady-state cruise power (encoded as *β = 3.2*). Smoother paths extend battery endurance, reduce charging frequency, and lower per-delivery energy consumption. As emphasized in recent research on UAM sustainability dimensions (Brunelli, 2022), energy efficiency is a critical metric for evaluating net environmental benefit alongside manufacturing footprint and end-of-life considerations.

Regulatory landscape. Our framework’s use of safety inflation (*Rsafe*) and geo-fencing-compatible waypoint planning is architecturally aligned with EASA Opinion 01/2020 (EASA, 2020) and FAA UTM v2.0 (FAA, 2020) separation requirements. However, BVLOS authorization demands demonstrated reliability under diverse conditions that simulation studies cannot fully establish. This regulatory gap represents a significant barrier to deployment.

Social acceptance and urban integration. Smoother paths with fewer sharp maneuvers reduce acoustic signature variation and increase obstacle clearance near pedestrian zones. Formal social acceptance studies and noise impact assessments are beyond the scope of this technical work; we direct future research in this direction (Brunelli, 2022). Technical performance improvements alone are insufficient for public acceptance of UAV logistics in urban environments.

## 6 Conclusion

This paper addressed the critical problem of path planning for low-altitude logistics UAVs in complex urban environments. Our central contribution is the principled integration of a multi-objective cost function—simultaneously penalizing path length, cumulative turning angle, and obstacle proximity risk—directly into the A* search phase, not merely as a post-hoc objective. This eliminates the architectural inconsistency present in all prior A*-based planners, where a length-only search objective is paired with a multi-objective declared goal.

The proposed hierarchical Search-and-Smooth framework delivers the following technical advances:

1. Objective-consistent search: Multi-objective edge cost *c(u,v) = α d + β Δθ + γ r* ensures the search phase genuinely minimizes *Jtotal.*
2. Provable efficiency: Weighted heuristic (*w = 1.5*) reduces computation by 42.8% with *≤ 2\%* empirical quality loss, provably bounded by w · C*.
3. Artifact elimination: Greedy LOS post-processor removes grid-discretization-induced turns, reducing cumulative yaw by 69.9% — equivalent to eliminating 4–5 full reversal maneuvers per mission.
4. Statistical rigor: Wilcoxon signed-rank tests (*N = 50*, *p < 0.001*) confirm that all improvements are statistically significant with large effect sizes.
5. Ablation-verified contributions: A dedicated ablation study (Table 1; Section 4.3.2) isolates the contribution of the multi-objective edge cost (+48.0% cumulative angle reduction alone) from LOS smoothing (+31.9% alone), confirming their synergistic combined effect (69.9% total reduction).
6. Competitive benchmarking: Comparison against Theta* and PSO confirms the proposed method’s competitive advantages in planning speed and geometric path quality within the tested synthetic simulation conditions.

The 69.9% reduction in cumulative turning angle translates directly to reduced ESC current stress, improved payload stability, and lower per-mission energy expenditure—since each eliminated heading-change maneuver avoids a differential-thrust energy spike. Quantifying the precise battery-life benefit requires hardware-in-the-loop validation with a specific airframe, which we identify as future work.

Scope and validation note. All quantitative results were obtained under controlled simulation conditions—a static 50 × 50 m synthetic map and a desktop workstation. These results establish the algorithmic properties of the proposed framework under those conditions, but should not be interpreted as evidence of operational readiness. Substantiating performance claims for real urban deployments requires hardware-in-the-loop testing, real-world map validation, dynamic obstacle scenarios, and regulatory compliance demonstration—all identified as future work.

Future work will extend the framework to: (a) 3D urban airspace with altitude-layer planning; (b) dynamic obstacle re-planning using a rolling-horizon approach; (c) multi-UAV fleet coordination with conflict resolution; (d) integration with Minimum Snap trajectory optimization as the downstream refiner of our LOS-smoothed waypoint skeleton; and (e) hardware-in-the-loop validation on embedded flight controllers to confirm real-time performance on deployment-realistic compute platforms.

## Data Availability

The original contributions presented in the study are included in the article/supplementary material, further inquiries can be directed to the corresponding author.
